# Characterization of the cluster MabR prophages of *Mycobacterium abscessus* and *Mycobacterium chelonae*

**DOI:** 10.1093/g3journal/jkac188

**Published:** 2022-07-27

**Authors:** Jacob Cote, Colin Welch, Madeline Kimble, Dakota Archambault, John Curtis Ross, Hector Orellana, Katelyn Amero, Claire Bourett, Andre Daigle, Keith W Hutchison, Sally D Molloy

**Affiliations:** Department of Molecular and Biomedical Sciences, University of Maine, Orono, ME 04469, USA; Department of Molecular and Biomedical Sciences, University of Maine, Orono, ME 04469, USA; The Honors College, University of Maine, Orono, ME 04469, USA; Department of Molecular and Biomedical Sciences, University of Maine, Orono, ME 04469, USA; The Honors College, University of Maine, Orono, ME 04469, USA; Department of Molecular and Biomedical Sciences, University of Maine, Orono, ME 04469, USA; The Honors College, University of Maine, Orono, ME 04469, USA; Department of Molecular and Biomedical Sciences, University of Maine, Orono, ME 04469, USA; Department of Molecular and Biomedical Sciences, University of Maine, Orono, ME 04469, USA; Department of Molecular and Biomedical Sciences, University of Maine, Orono, ME 04469, USA; The Honors College, University of Maine, Orono, ME 04469, USA; Department of Molecular and Biomedical Sciences, University of Maine, Orono, ME 04469, USA; The Honors College, University of Maine, Orono, ME 04469, USA; Department of Molecular and Biomedical Sciences, University of Maine, Orono, ME 04469, USA; Department of Molecular and Biomedical Sciences, University of Maine, Orono, ME 04469, USA; The Honors College, University of Maine, Orono, ME 04469, USA; Department of Molecular and Biomedical Sciences, University of Maine, Orono, ME 04469, USA; The Honors College, University of Maine, Orono, ME 04469, USA

**Keywords:** prophage, *Mycobacterium*, bacteriophage, genome

## Abstract

*Mycobacterium abscessus* is an emerging pathogen of concern in cystic fibrosis and immunocompromised patients and is considered one of the most drug-resistant mycobacteria. The majority of clinical *Mycobacterium abscessus* isolates carry 1 or more prophages that are hypothesized to contribute to virulence and bacterial fitness. The prophage McProf was identified in the genome of the Bergey strain of *Mycobacterium chelonae* and is distinct from previously described prophages of *Mycobacterium abscessus*. The McProf genome increases intrinsic antibiotic resistance of *Mycobacterium chelonae* and drives expression of the intrinsic antibiotic resistance gene, *whi*B7, when superinfected by a second phage. The prevalence of McProf-like genomes was determined in sequenced mycobacterial genomes. Related prophage genomes were identified in the genomes of 25 clinical isolates of *Mycobacterium abscessus* and assigned to the novel cluster, MabR. They share less than 10% gene content with previously described prophages; however, they share features typical of prophages, including polymorphic toxin–immunity systems.

## Introduction

Prophages are viral genomes integrated into bacterial genomes and they contribute to the genetic diversity and virulence of many bacterial pathogens (Figueroa‐Bossi *et al.* 2001; Brüssow *et al.* 2004; Fortier and Sekulovic 2013; Wang and Wood 2016; Costa *et al.* 2018; Fortier 2018). Clinically important nontuberculosis mycobacteria (NTM), such as *Mycobacterium abscessus*, often cause drug-resistant infections and continue to be a significant public health burden (Nasiri *et al.* 2017). The majority of clinical NTM carry prophage genomes that are enriched in genes that potentially promote bacterial fitness and virulence (Glickman *et al.* 2020; Dedrick *et al.* 2021).

The prophages of *M. abscessus* are vastly diverse and distinct from the mycobacteriophage genomes in the Actinobacteriophage database of phagesdb.org (Russell and Hatfull 2017; Dedrick *et al.* 2021). Dedrick *et al.* (2021) identified 122 prophage sequences in 82 clinical isolates of *M. abscessus* of which 67 were unique. These were sorted into 17 Mab clusters (MabA—MabQ) based on the shared gene content (>35% shared genes) (Dedrick *et al.* 2021). Many of the prophages encode toxin/antitoxin and polymorphic toxin–immunity (PT-Imm) systems that are hypothesized to contribute to virulence (Zhang *et al.* 2012; Dedrick *et al.* 2021). We recently described a novel prophage genome, named McProf, in the genome of *Mycobacterium chelonae* (*M. chelonae* CCUG 47445 coordinates 1,521,426–1,589,648) that shares only 10% gene content with the Dedrick *et al.* prophages but encodes numerous genes expressed during lysogeny, including a PT-Imm system (Cushman *et al.* 2021). McProf contributes to the intrinsic drug resistance of *M. chelonae* and increases expression of the conserved mycobacterial regulator of intrinsic antibiotic resistance genes, *whi*B7, when superinfected by a second mycobacteriophage (Cushman *et al.* 2021). Understanding the prevalence of this novel prophage genome and its relationship with known prophage genomes will be important for a better understanding of the role of prophage genomes in mycobacterial fitness and virulence.

In this study, prophage genomes related to McProf were identified in 25 published genomes of *M. abscessus*, and in 1 genome of *Mycobacterium phlei*. Gene content was compared with prophage genomes described by Dedrick *et al.* (2021) and sorted into a novel cluster, MabR (Dedrick *et al.* 2021). Here, we report the genomes of 5 unique cluster MabR genomes, including 4 *M. abscessus* prophages and the original *M. chelonae* prophage McProf.

## Materials and methods

### Identification and extraction of prophage from mycobacterial genomes

Prophage sequences similar to McProf were identified using the PhagesDB BLASTn tool to search *M. abscessus* genomes within the PATRIC database (Altschul *et al.* 1990; Wattam *et al.* 2014; Russell and Hatfull 2017). High scoring sequences were analyzed using PHASTER to determine the putative coordinates of prophage genomes within bacterial genome sequences (Arndt *et al.* 2016). Precise coordinates were determined after manual inspection of prophage genomes and identification of repeat sequences that flank the prophage genome and represent the common core of *attL*/*attR* sites. Each prophage sequence was extracted with the identified attachment sites defining the genome ends. Prophages were named according to the strain in which they reside, i.e. prophiXXXX01-1, with suffixes used to denote multiple prophages in the same genome as described by Dedrick *et al.* (2021).

### Prophage genome annotation and comparative genomics

Prophage genes were predicted using Glimmer and GeneMark within DNA Master (http://cobamide2.bio.pitt.edu/) and PECAAN (https://discover.kbrinsgd.org/) (Delcher *et al.* 1999; Borodovsky *et al.* 2003). The start site for each gene was determined through manual inspection. Gene functions were predicted using the web-based tools HHpred and NCBI BLASTp (Altschul *et al.* 1990; Söding *et al.* 2005). Dot plots were constructed using gepard using default settings (Krumsiek *et al.* 2007). The prophage network phylogeny is based on shared gene content and was created in SplitsTree (Huson 1998). Genome maps were created using Phamerator and the “Actino_Mab_Draft” database, version 19 (Cresawn *et al.* 2011). Integration sites were predicted by comparing flanking bacterial sequence in each prophage genome to that of *M. abscessus* ATCC 19977. Specific integration locations were determined by probing the previous integration region with the *attL* sequence for each prophage. Alignments with 100% sequence identity were considered to be core *attB* sites.

## Results

### Identification of cluster MabR prophage

To identify prophage sequences related to the *M. chelonae* prophage McProf, we searched the NCBI database using BLASTN and identified a prophage sequence in the *M. phlei* strain NCTC8151 (accession number LR134347) with 100% nucleotide identity to the McProf genome. To search for McProf-like sequences in *M. abscessus* genomes, we probed the PATRIC database with the McProf genome sequence using the BLASTN feature within phagesdb.org (Altschul *et al.* 1990; Wattam *et al.* 2014; Russell and Hatfull 2017). We identified 25 *M. abscessus* clinical strains carrying prophage sequences with high sequence identity (greater than 70% across the majority of the genome) to the McProf genome (Table 1). All of the *M. abscessus* strains were isolated from the respiratory system of diseased individuals, and the vast majority of the *M. abscessus* strains were isolated in the United Kingdom (76%) (Table 1). The remaining 24% of strains were isolated in the United States (16%) and Australia (8%).

**Table 1.** *M. abscessus* bacterial strains carrying MabR prophage.

	Accession no.^a^	**MabR prophage** ^b^ ^,^ ^c^	**Coordinates** ^d^ ^,^ ^e^	Additional prophage^b^	Coordinates^d^	**Origin** ^f^	Subspecies	Strain
FSAT01	GCA_900131665.1	prophiFSAT01-1	C1 2,104,368–2,172,096	–	–	United Kingdom	*abscessus*	280
FSIL01	GCA_900136245.1	prophiFSIL01-1	C6 162,543–229,039	prophiFSIL01-2 (MabA1)	C2 491,511–553,312	United Kingdom	*abscessus*	1,009
FSGY01	GCA_900135415.1	prophiFSIL01-1	C4 326,208–259,712	prophiFSIL01-2 (MabA1)	C2 209,626–147,825	United Kingdom	*abscessus*	62
FSGZ01	GCA_900135455.1	prophiFSIL01-1	C7 228,809–162,313	prophiFSIL01-2 (MabA1)	C2 491,649–553,450	United Kingdom	*abscessus*	63
FSHA01	GCA_900135465.1	prophiFSIL01-1	C6 228,812–162,316	prophiFSIL01-2 (MabA1)	C2 209,313–147,512	United Kingdom	*abscessus*	64
FSHB01	GCA_900135495.1	prophiFSIL01-1	C2 229,051–162,555	prophiFSIL01-2 (MabA1)	C2 563,712–501,911	United Kingdom	*abscessus*	314
FSHC01	GCA_900135485.1	prophiFSIL01-1	C6 125,119–191,615	prophiFSIL01-2 (MabA1)	C3 209,298–147,497	United Kingdom	*abscessus*	66
FSHD01	GCA_900135515.1	prophiFSIL01-1	C7 125,118–191,614	prophiFSIL01-2 (MabA1)	C3 491,671–553,472	United Kingdom	*abscessus*	67
FSHE01	GCA_900135475.1	prophiFSIL01-1	C7 125,120–191,616	prophiFSIL01-2 (MabA1)	C2 209,310–147,509	United Kingdom	*abscessus*	68
FSHF01	GCA_900135505.1	prophiFSIL01-1	C1 826,179–162,313	prophiFSIL01-2 (MabA1)	C1 491,510–553,311	United Kingdom	*abscessus*	69
FSHG01	GCA_900135535.1	prophiFSIL01-1	C6 228,810–162,314	prophiFSIL01-2 (MabA1)	C2 479,152–417,351	United Kingdom	*abscessus*	70
FSHI01	GCA_900135525.1	prophiFSIL01-1	C7 228,802–162,306	prophiFSIL01-2 (MabA1)	C3 209,315–147,514	United Kingdom	*abscessus*	71
FSIG01	GCA_900136185.1	prophiFSIL01-1	C5 228,798–162,543	prophiFSIL01-2 (MabA1)	C3 491,467–553,268	United Kingdom	*abscessus*	991
FSIH01	GCA_900136155.1	prophiFSIL01-1	C1 1,678,669–1,745,165	prophiFSIL01-2 (MabA1)	C2 707,577–769,378	United Kingdom	*abscessus*	993
FSIJ01	GCA_900136115.1	prophiFSIL01-1	C6 125,125–191,621	prophiFSIL01-2 (MabA1)	C3 209,291–147,490	United Kingdom	*abscessus*	996
FSIQ01	GCA_900136355.1	prophiFSIL01-1	C6 228,795–162,299	prophiFSIL01-2 (MabA1)	C2 491,560–553,361	United Kingdom	*abscessus*	1,019
FSKF01	GCA_900137275.1	prophiFSIL01-1	C5 125,178–191,674	prophiFSIL01-2 (MabA1)	C2 491,625–553,426	United Kingdom	*abscessus*	1,024
FVMH01	GCA_900136085.1	prophiFSIL01-1	C6 228,779–162,283	prophiFSIL01-2 (MabA1)	C1 1,082,839–1,144,640	United Kingdom	*abscessus*	994
FVPC01	GCA_900137305.1	prophiFSIL01-1	C1 544,352–477,856	prophiFSIL01-2 (MabA1)	C1 879,057–817,256	United Kingdom	*abscessus*	1,026
FSQJ01	GCA_900141335.1	prophiFSQJ01-1	C10 102,082–169,883	prophiFSQJ01-3 (MabD)	C12 50,449–101,334	United States	*abscessus*	712
FSMS01	GCA_900139245.1	prophiFSQJ01-1	C13 99,951–167,702	prophiFSMS01-2 (MabD),	C7 85,005–135,096	United States	*abscessus*	699
				prophiFSMS01-3 (MabG)	C7 156,631–209,932			
FSOD01	GCA_900140065.1	prophiFSQJ01-1	C13 85,252–17,501	–	–	United States	*abscessus*	686
FVXT01	GCA_900141255.1	prophiFSQJ01-1	C10 93,905–26,154	prophiFSMS01-2 (MabD)	C7 84,991–135,082	United States	*abscessus*	698
				prophiFSMS01-3 (MabG)	C7 156,617–209,918			
FVLO01	GCA_900135885.1	prophiFVLQ01-1	C1 163,540–230,227	prophiFVLQ01-2 (MabD),	C15 73,430–126,907	Australia	*bolletii*	874
				prophiFVLQ01-3 (MabC)	C5 363,193–311,358			
FVLQ01	GCA_900135895.1	prophiFVLQ01-1	C2 360,992–427,679	prophiFVLQ01-2 (MabD),	C4 73,988–127,465	Australia	*bolletii*	875
				prophiFVLQ01-3 (MabC)	C7 510–52,345			

a GenBank assembly accession numbers.

b Prophages are named after the first genome where they were first isolated, identical prophage in other genomes use the same name.

c MabR prophage in the genomes FSGY01, FSGZ01, FSHE01, FSHG01, FVHM01, FSMS01, and FVXT01 have single-nucleotide differences with their representative prophage genome.

d The contig number (C1, C2, etc.) is shown followed by the coordinates within that contig.

e MabR prophage coordinates in representative host genomes (FSAT01, FSIL01, FSQJ01, and FVLQ01) are ordered from attL to attR.

f All genome samples were isolated from the respiratory system of diseased hosts in the country indicated.

**Fig. 1.**
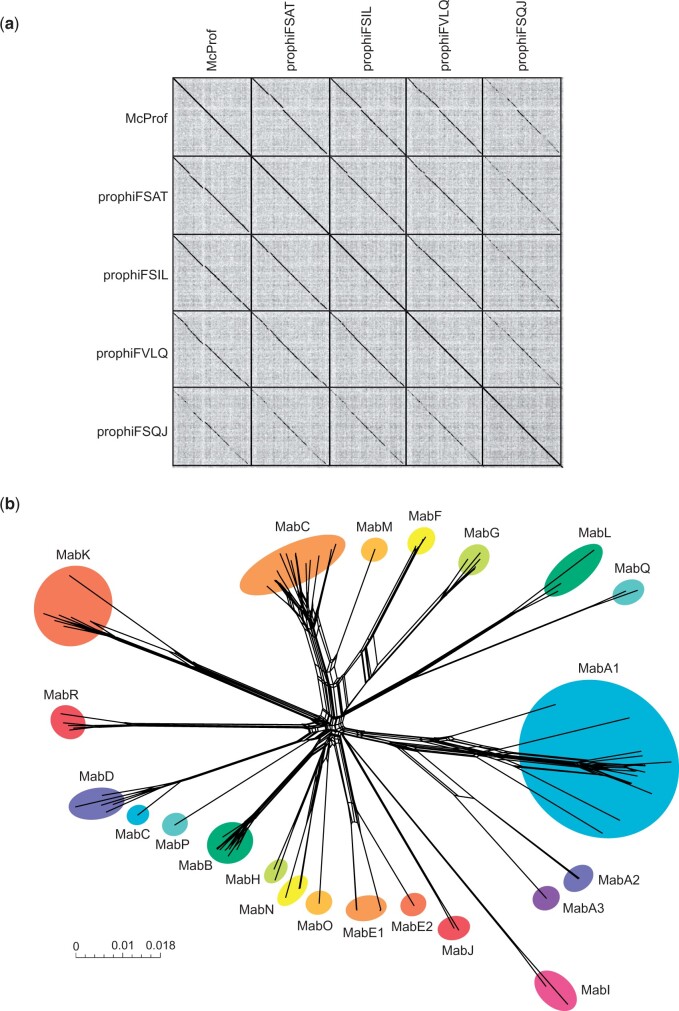
Diversity of MabR prophages. a) Dotplot comparison of MabR prophages. b) Phylogenetic network representation of cluster MabR prophages and *M. abscessus* prophages (Dedrick *et al.* 2021) based on shared gene content as described by Pope *et al*. (2015). Nodes represent individual prophage; circles represent prophage clusters. Scale marker indicates substitutions/site.

Of the 25 identified McProf-like prophage sequences in *M. abscessus*, only 4 prophage sequences were unique. Strains carrying identical prophage sequences are indicated in Table 1. The 4 unique prophage sequences were extracted from the bacterial sequences of the following *M. abscessus* strains: FSAT01, FSIL01, FSQJ01, and FVLQ01 (Table 2). The ends of the prophage genomes were determined by the left and right attachment sites flanking the prophage genomes (Table 2). Prophages were named by the strain they were extracted from and the number of prophages identified in the strain: prophi[strain]-# (Table 2). McProf and the 4 McProf-like prophage genomes: prophiFSAT01-1, prophiFSIL01-1, prophiFSQJ01-1, and prophiFVLQ01-1 share less than 10% genome content with the *M. abscessus* prophages described by Dedrick *et al.* (2021) and were assigned to a novel cluster, MabR (Fig. 1a) (Dedrick *et al.* 2021). The MabR prophages overall have high nucleotide similarity to one another (Fig. 1b).

**Table 2.** Genome characteristics of cluster MabR prophages.

Prophage	***att*B** ^a^	Coordinates^b^	Length^c^	ORFs	*att*L/R sequences	Accession
McProf^d^	*att*B-18	1,521,426–1,589,648	68,223	99	TGCGCCGTCAGGGGCTCGAACCCCGGACCCGCTGATTAAGAGTCA	BK061309
prophiFSAT01-1	*att*B-18	C1 2,104,368–2,172,096	67,729	99	TGCGCCGTCAGGGGCTCGAACCCCGGACCCGCTGATTAAGAGTCA	BK061308
prophiFSIL01-1	*att*B-22	C6 162,543–229,039	66,497	99	TGCGCCGTCAGGGTTTCGAACCCCAGACCCGCTGATTAAGAGTCA TGCGCCGTCAGGGGCTCGAACCCCGGACCCGCTGATTAAGAGTCA	BK061311
prophiFSQJ01-1	*att*B-23	C10 102,082–169,883	67,752	102	CCCCTGTAGGGCTCGAACCTACGACCTACTGATTAAAAGTCAG CCCCACCAGGGCTCGAACCTGGGACCTGCGGATTAAAAGTCCG	BK061312
prophiFVLQ01-1	*att*B-18	C2 360,992–427,679	66,688	100	TGACTCTTAATCAGCGGGTCCGGGGTTCGAGCCCCTGACGGCGCA	BK061310

a *att*B-18 was identified by Dedrick *et al.* (2021).

b Coordinates of the selected phage in the host where it was first identified (e.g. prophiFSAT01-1 in the genome FSAT01). The contig number (C1, C2, etc.) is shown followed by the coordinates within that contig. Coordinates are arranged *att*L to *att*R.

c Prophage lengths include 2 copies of the attachment sites.

d McProf is a previously described prophage (Cushman *et al.* 2021) found in the *M. chelonae* genome CCUG 47445.

### Integration locations

**Fig. 2.**
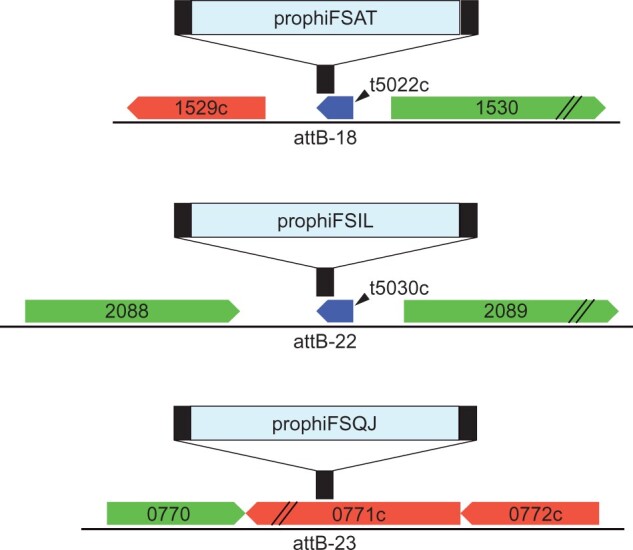
MabR prophage integration locations. The 3 integration schemes used by MabR prophage are shown as *attB* site locations (black bars) shown relative to *M. abscessus* ATCC 19977 genes for reference. Rightward and leftward transcribed genes are indicated by arrows with their ATCC 19977 gene number. Both tRNAs (t5022c and t5030c) are transcribed in the leftward direction. Not shown are McProf and prophiFVLQ01-1, which utilize the *attB-18* site described by Dedrick *et al.* (2021).

The integration sites of MabR prophage were determined and compared to that of prophage described by Dedrick *et al.* (2021). The prophage genomes integrated into known *M. abscessus attB* sites, often in the 3′ end of tRNA genes (Table 3). Three prophage genomes, McProf, prophiFSAT01-1, and prophiFVLQ01-1, integrate into the 3′ end of a tRNA-Lys (*attB*-18) as described in Dedrick *et al.* (2021) (Fig. 2). prophiFSIL01-1 integrates into the 3′ end of a tRNA-Lys (*attB*-22) and prophiFSQJ01-1 integrates into Mab_0771c (*attB*-23), a predicted major transport protein. *attB*-23 was the only cluster MabR integration site identified within a protein-coding sequence. The *attB* core sequences and coordinates for each identified integration site are listed in Table 3.

**Table 3.** attB sites of cluster MabR prophage.

*att*B^a^	Core sequence^b^	Prophages^c^	Genomic feature^d^	Coordinates^d^
*att*B-18	CTGGTGCGCCGTCAGGGGCTCGAACCCCGGACCCGCTGATTAAGAGTC	McProf, prophiFSAT01-1, prophiFVLQ01-1	Mab_t5022c; 3′ end tRNA-Lys	1,550,157–1,550,204
*att*B-22	TGCGCCGTCAGGG**TT**TCGAACCCC**A**GACCCGCTGATTAAGAGTCA	prophiFSIL01-1	Mab_t5030c; 3′ half of tRNA-Lys	2,089,033–2,089,077
*att*B-23	CCCC**ACC**AGGGCTCGAACCT**GG**GACCT**G**C**G**GATTAAAAGTC**CG**	prophiFSQJ01-1	In Mab_0771c	770,355–770,397

a *att*B-18 was identified by Dedrick *et al.* (2021).

b Sequence shared between *att*L and *att*R sites within and near the genomic feature for each *att*B site; mismatches are shown in bold. Novel *att*B sites (*att*B 19, 20) have no mismatches when aligning to *att*R sites in their respective phage.

c As defined in Table 2.

d Genes and coodinates in the *M. abscessus* strain ATCC1997.

### Genomic organization of cluster MabR genomes

**Fig. 3.**
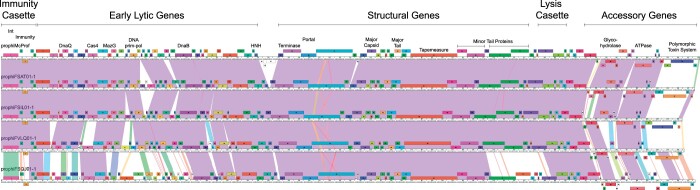
Organization of MabR genomes. MabR genomes are shown with pairwise nucleotide sequence similarity displayed by colors between genomes: purple is the most similar and red is the least similar above a BLASTN E threshold of 10^−5^. The ruler represents the coordinates of the genome. Forward and reverse-transcribed genes are shown as boxes above and below the ruler, respectively. Maps were generated using Phamerator and the database, “Actino_Mab_Draft (version 20)” (Cresawn *et al.* 2011).

**Fig. 4.**
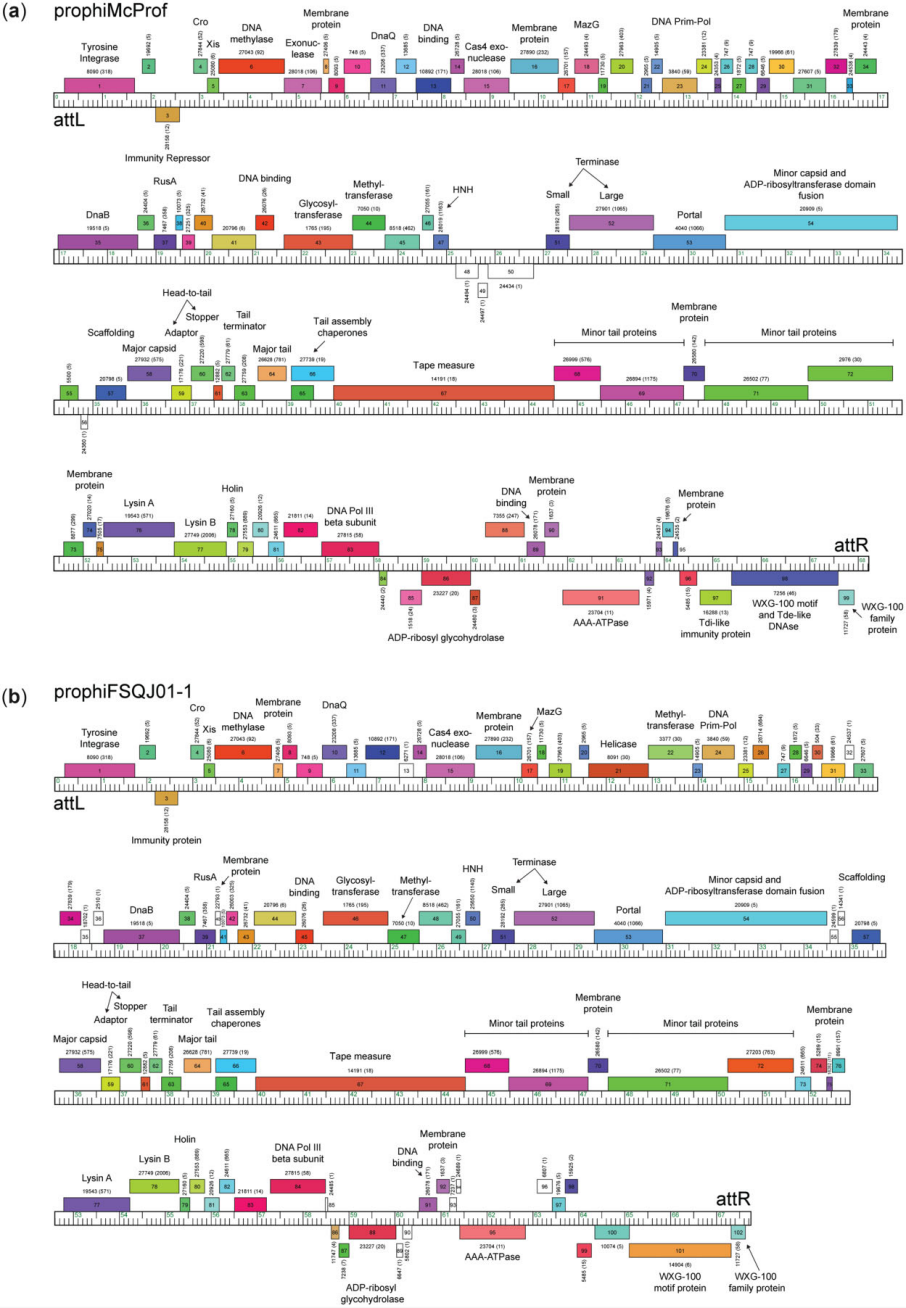
Genome organization of a) prophiMcProf and b) prophiFSQJ01-1. The ruler represents the coordinates of the genome. Forward and reverse-transcribed genes are shown as boxes above and below the ruler, respectively. Genes are colored according to their assigned Phamily with the Phamily number shown above each gene with the number of Phamily members in parentheses. Genome maps were generated using Phamerator and the database, “Actino_Mab_Draft (version 20)” (Cresawn *et al.* 2011).

MabR prophages have very similar genome architectures and areas of conserved gene content (Fig. 3). The genomes are tightly packed, typical of mycobacteriophage genomes, containing 98–102 genes across approximately 67 kb. The integration and immunity cassettes are located immediately adjacent to the left attachment site (*attL*). All MabR genomes share a rightward transcribed tyrosine integrase (gp1), a gene of unknown function (gp2), and a leftward transcribed phage repressor (gp3) (superinfection immunity repressor) (Figs. 3, 4 and S1). The immunity repressor is distinct from immunity repressors encoded by other Mab cluster prophages; however, it is a homolog of the immunity repressors found in the genomes of 5 cluster K2 mycobacteriophage, DismalFunk, DismalStressor, Findely, Marcoliusprime, and Milly. A Cro and excise gene (gp4 and 5) are divergently transcribed from the immunity repressor (Figs. 3 and 4). The early lytic genes that follow show some diversity across the 5 MabR genomes, particularly in prophiFSQJ01-1. The structural, assembly, and lysis cassette genes are highly conserved across MabR genomes.

**Fig. 5.**
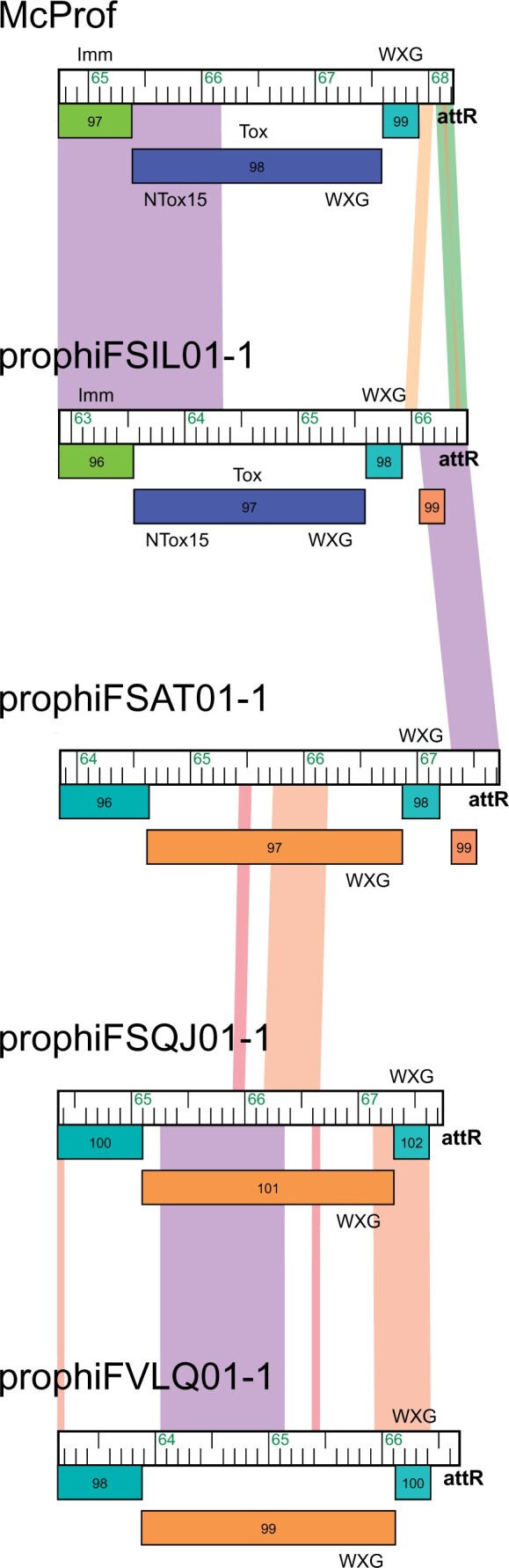
Organization of MabR PT-Imm systems. MabR genomes are aligned at their PT-Imm systems beginning at the 3′ end of the predicted immunity proteins. Genomes are displayed as described in Fig. 2 but are ordered in such a way that genomes with the most similarity in this region are next to each other. Also shown are the motifs/domains found at the N- and C-termini of MabR PTs. All predicted PTs have a single WXG-100 motif at the N-terminus while the C-terminus is variable. Note that gp99 in prophiFSIL01-1 and prophiFSAT01-1 has no predicted function and is included to show the relationship of the PT systems to the genome ends.

Between the lysis cassette and the right attachment site (*attR*) is a group of diverse genes that are most likely expressed during lysogeny (Fig. 3) (Dedrick *et al.* 2017; Cushman *et al.* 2021). Some of the genes shared across all MabR genomes are unique to the cluster and include a DNA polymerase III sliding clamp, an ADP-ribosyl glycohydrolase, a helix-turn-helix DNA binding domain protein, and an AAA-ATPase. Immediately adjacent to *attR*, all MabR prophage genomes encode a reverse-transcribed PT-Imm system that include an ESAT6-like WXG-100 protein, a polymorphic toxin (PT), and cognate immunity protein (Figs. 3 and 5).

### Polymorphic toxin systems

**Fig. 6.**
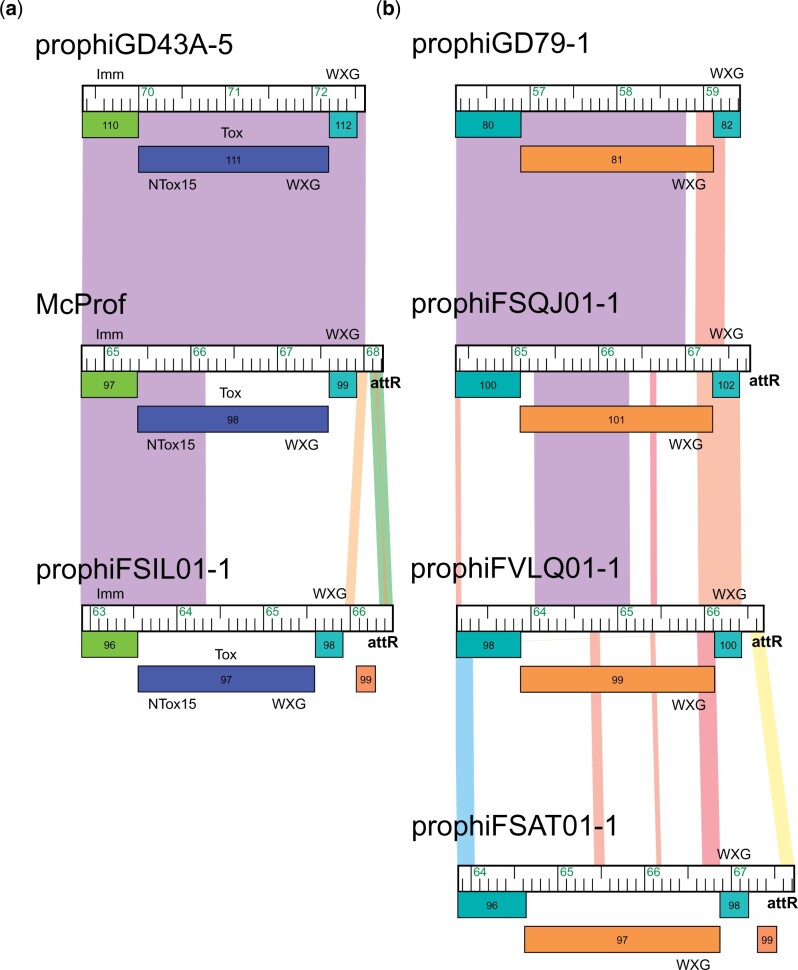
MabR PT-Imm systems found in non-MabR prophage. Genomes are displayed as described in Figs. 2 and 5. prophiGD43-5 and prophiGD79-1 belong to clusters MabK and MabQ, respectively.

Dedrick *et al.* (2021) identified 21 distinct, modular, PT-Imm systems across 50 *M. abscessus* prophage (Dedrick *et al.* 2021). These systems consist of a large PT and a cognate immunity protein (PT-Imm) to prevent self-toxicity and at least 1 ESAT6-like WXG-100 protein. The cluster MabR genomes contain one of 2 types of PT-Imm systems (Figs. 3 and 5). The PT in the McProf and prophiFSIL01-1 genomes has an N-terminus WXG-100 domain and a C-terminus Tde-like DNAse toxin domain (Ntox15 PF15604) (Ma *et al.* 2014; Cushman *et al.* 2021). Downstream is the Tdi-like PT-Imm protein with GAD-like and DUF1851 domains (Ma *et al.* 2014). This PT-Imm system is also found in the genome of prophiGD43A-5 (Fig. 6). Although the 3 PT genes carry the same Ntox15 domain, they share low sequence identity across the linker and WXG-100 domains. In the NCBI database, this PT-Imm system is also found in the genomes of *Mycobacterium* phage phiT46-1 (accession number NC_054432.1) and numerous mycobacterial species including *M. abscessus*, *Mycobacterium goodie*, and *Mycobacterium salmoniphilum*.

The genomes of prophiFSAT01-1, prophiFSQJ01-1, and prophiFVLQ01-1 carry a gene cassette that is organized like a PT-Imm system and encodes an ESAT6-like WXG-100 protein (Fig. 5). However, we were unable to predict toxin and immunity domains. The presumed PT gene has an N-terminus WXG-100 domain but lacks an identifiable toxin domain in the C-terminus. Likewise, the downstream gene lacks domains known to be associated with immunity, such as SUKH or Imm (Zhang *et al.* 2012; Dedrick *et al.* 2021). This second PT-Imm system is also found in the cluster MabQ genome, prophiGD79-1 (Fig. 6) (Dedrick *et al.* 2021).

## Discussion

The majority of bacterial pathogens carry prophages that are known to contribute to bacterial virulence and fitness (Figueroa-Bossi *et al.* 2001; Brüssow *et al.* 2004; Wang and Wood 2016). Prophage introduces novel genes into bacterial genomes that can result in phenotypes that are more competitive in bacterial populations (Brüssow *et al.* 2004; Wang and Wood 2016). The prophage McProf is found in the Bergey strain of *M. chelonae* (ATCC 35752) and increases bacterial resistance to aminoglycosides (Cushman *et al.* 2021). Although the McProf genome is distinct from the *M. abscessus* prophages described by Dedrick *et al.* (2021) (Dedrick *et al.* 2021), it is clearly related to a novel subgroup of prophage genomes identified in the genomes of clinical *M. abscessus* isolates and, therefore, was assigned to the novel cluster, MabR.

The majority of the MabR prophages were identified in the genomes of *M. abscessus* isolates, although a prophage genome that shared 100% nucleotide with McProf was identified in *M. phlei*. Of the 25 MabR genomes identified in *M. abscessus* strains, only 4 were unique and these were typically found in isolates with the same geographical origin (Table 1). Strains of the same geographic origin also typically carried identical cohabitating prophages, suggesting that the bacterial strains are highly related.

The MabR prophage genomes, although distinct in overall gene content, share a genome organization and some gene features that are typical of the prophages described by Dedrick *et al.* (2021) (Dedrick *et al.* 2021). These include 2 types of PT-Imm systems that potentially contribute to mycobacterial fitness (Figs. 5 and 6) (Zhang *et al.* 2012). The PT-Imm systems of McProf and prophiFSIL01-1 are similar to the PT-Imm system that plays a role in plant colonization in the Gram-negative plant pathogen, *Agrobacterium tumefaciens* (Ma *et al.* 2014). The PTs share a C-terminal DNAse toxin domain (Ntox-15) but differ at the N-terminal domain, which contains a WXG100 domain needed for interacting with type VII secretion systems (T7SS) in mycobacteria vs the PAAR domain needed for type VI secretion systems in *Agrobacterium* (Ma *et al.* 2014). It is not yet known whether mycobacterial prophage-encoded toxins are secreted, but it is hypothesized that the toxin dimerizes with the small WXG-100 protein (gp99 in McProf) via the WXG100 domains and is secreted by the mycobacterial T7SS (Esx-3 or Esx-4) (Zhang *et al.* 2012; Cushman *et al.* 2021; Dedrick *et al.* 2021).

It is not clear yet if the PT-Imm systems of the MabR prophage are important for bacterial fitness, but it is known that the presence of the McProf genome increases *M. chelonae* resistance to aminoglycosides relative to a nonlysogen strain (Cushman *et al.* 2021). The addition of a second prophage, cluster G phage BPs, to this strain further increased the aminoglycoside resistance and increased the expression of mycobacterial antibiotic resistance genes in the *whi*B7 regulon, including *whi*B7 (Sampson *et al.* 2009; Cushman *et al.* 2021). This large change in *whi*B7 expression and aminoglycoside resistance is driven by the presence of the McProf genome as it is not observed in strains carrying the BPs’ prophage alone. There are 16 genes expressed from the McProf genome during lysogeny of *M. chelonae* that potentially contribute to altered *whi*B7 expression and increased aminoglycoside expression (Cushman *et al.* 2021). Many of these genes are common across the MabR genomes including the McProf PT-Imm cassette (gp97–99), gp91 and 92, and gp85 and 86 (Fig. 3). A better understanding of the function and role these genes potentially play in mycobacterial fitness will improve our overall understanding of how prophage contributes to mycobacterial virulence.

## Data availability

The bacterial genome coordinates of the MabR prophage genomes and the bacterial genome accession numbers are presented in Table 1. The genome sequences and annotations of prophages McProf (accession no. BK061309), prophiFSAT01-1 (accession no. BK061308), prophiFSIL01-1 (accession no. BK061311), prophiFVLQ01-1 (accession no. BK061310), and prophiFSQJ01-1 (accession no. BK061312) are available through NCBI GenBank.

Supplemental material is available at *G3* online.

## Supplementary Material

jkac188_Supplementary_Figure_Legend

jkac188_Supplementary_FigureS1

## References

[jkac188-B1] Altschul SF , Gish W, Miller W, Myers EW, Lipman DJ. Basic local alignment search tool. J Mol Biol. 1990;215(3):403–410. 2231712 10.1016/S0022-2836(05)80360-2

[jkac188-B2] Arndt D , Grant JR, Marcu A, Sajed T, Pon A, Liang Y, Wishart DS. Phaster: a better, faster version of the phast phage search tool. Nucleic Acids Res. 2016;44(W1):W16–W21. 27141966 10.1093/nar/gkw387 PMC4987931

[jkac188-B3] Borodovsky M , Mills R, Besemer J, Lomsadze A. Prokaryotic gene prediction using GeneMark and GeneMark.hmm. Curr Protoc Bioinformatics. 2003;Chapter 4:Unit4.5. 10.1002/0471250953.bi0405s01 18428700

[jkac188-B4] Brüssow H , Canchaya C, Hardt W-D. Phages and the evolution of bacterial pathogens: from genomic rearrangements to lysogenic conversion. Microbiol Mol Biol Rev. 2004;68(3):560–602. 15353570 10.1128/MMBR.68.3.560-602.2004 PMC515249

[jkac188-B5] Costa AR , Monteiro R, Azeredo J. Genomic analysis of acinetobacter baumannii prophages reveals remarkable diversity and suggests profound impact on bacterial virulence and fitness. Sci Rep. 2018;8(1):15346. 30337588 10.1038/s41598-018-33800-5 PMC6193963

[jkac188-B6] Cresawn SG , Bogel M, Day N, Jacobs-Sera D, Hendrix RW, Hatfull GF. Phamerator: a bioinformatic tool for comparative bacteriophage genomics. BMC Bioinformatics. 2011;12(1):395. 21991981 10.1186/1471-2105-12-395 PMC3233612

[jkac188-B7] Cushman J , Freeman E, McCallister S, Schumann A, Hutchison KW, Molloy SD. Increased whib7 expression and antibiotic resistance in *Mycobacterium chelonae* carrying two prophages. BMC Microbiol. 2021;21(1):176. 34107872 10.1186/s12866-021-02224-z PMC8191103

[jkac188-B8] Dedrick RM , Aull HG, Jacobs-Sera D, Garlena RA, Russell DA, Smith BE, Mahalingam V, Abad L, Gauthier CH, Hatfull GF. The prophage and plasmid mobilome as a likely driver of *Mycobacterium abscessus* diversity. mBio. 2021;12(2):e03441-20. 33785627 10.1128/mBio.03441-20 PMC8092301

[jkac188-B9] Dedrick RM , Jacobs-Sera D, Bustamante CAG, Garlena RA, Mavrich TN, Pope WH, Reyes JCC, Russell DA, Adair T, Alvey R, et al Prophage-mediated defence against viral attack and viral counter-defence. Nat Microbiol. 2017;2(3):16251. 28067906 10.1038/nmicrobiol.2016.251 PMC5508108

[jkac188-B10] Delcher AL , Harmon D, Kasif S, White O, Salzberg SL. Improved microbial gene identification with glimmer. Nucleic Acids Res. 1999;27(23):4636–4641. 10556321 10.1093/nar/27.23.4636 PMC148753

[jkac188-B11] Figueroa‐Bossi N , Uzzau S, Maloriol D, Bossi L. Variable assortment of prophages provides a transferable repertoire of pathogenic determinants in *Salmonella*. Mol Microbiol. 2001;39(2):260–272. 11136448 10.1046/j.1365-2958.2001.02234.x

[jkac188-B12] Fortier L-C. Bacteriophages contribute to shaping *Clostridioides (Clostridium) difficile* species. Front Microbiol. 2018;9:2033–2033. 30233520 10.3389/fmicb.2018.02033 PMC6127314

[jkac188-B13] Fortier L-C , Sekulovic O. Importance of prophages to evolution and virulence of bacterial pathogens. Virulence. 2013;4(5):354–365. 23611873 10.4161/viru.24498 PMC3714127

[jkac188-B14] Glickman C , Kammlade SM, Hasan NA, Epperson LE, Davidson RM, Strong M. Characterization of integrated prophages within diverse species of clinical nontuberculous mycobacteria. Virol J. 2020;17(1):1–13. 32807206 10.1186/s12985-020-01394-y PMC7433156

[jkac188-B15] Huson DH. SplitsTree: analyzing and visualizing evolutionary data. Bioinformatics (Oxford, England). 1998;14(1):68–73. 10.1093/bioinformatics/14.1.68 9520503

[jkac188-B16] Krumsiek J , Arnold R, Rattei T. Gepard: a rapid and sensitive tool for creating dotplots on genome scale. Bioinformatics. 2007;23(8):1026–1028. 17309896 10.1093/bioinformatics/btm039

[jkac188-B17] Ma L-S , Hachani A, Lin J-S, Filloux A, Lai E-M. *Agrobacterium tumefaciens* deploys a superfamily of type VI secretion DNase effectors as weapons for interbacterial competition in planta. Cell Host Microbe. 2014;16(1):94–104. 24981331 10.1016/j.chom.2014.06.002 PMC4096383

[jkac188-B18] Nasiri MJ , Haeili M, Ghazi M, Goudarzi H, Pormohammad A, Imani Fooladi AA, Feizabadi MM. New insights in to the intrinsic and acquired drug resistance mechanisms in mycobacteria. Front Microbiol. 2017;8:681. 28487675 10.3389/fmicb.2017.00681 PMC5403904

[jkac188-B4689115] Pope WH, , Bowman CA, , Russell DA, , Jacobs-Sera D, , Asai DJ, , Cresawn SG, , Jacobs WR, , Hendrix RW, , Lawrence JG, , Hatfull GF. Whole genome comparison of a large collection of mycobacteriophages reveals a continuum of phage genetic diversity. eLife. 2015;4. doi:10.7554/eLife.06416 PMC4408529 25919952

[jkac188-B20] Russell DA , Hatfull GF. PhagesDB: the actinobacteriophage database. Bioinformatics. 2017;33(5):784–786. 28365761 10.1093/bioinformatics/btw711 PMC5860397

[jkac188-B21] Sampson T , Broussard GW, Marinelli LJ, Jacobs-Sera D, Ray M, Ko CC, Russell D, Hendrix RW, Hatfull GF. Mycobacteriophages BPs, Angel and Halo: comparative genomics reveals a novel class of ultra-small mobile genetic elements. Microbiology (Reading). 2009;155(Pt 9):2962–2977. 19556295 10.1099/mic.0.030486-0 PMC2833263

[jkac188-B22] Söding J , Biegert A, Lupas AN. The HHpred interactive server for protein homology detection and structure prediction. Nucleic Acids Res. 2005;33(Web Server issue):W244–W248. 15980461 10.1093/nar/gki408 PMC1160169

[jkac188-B23] Wang X , Wood TK. Cryptic prophages as targets for drug development. Drug Resist Updat. 2016;27:30–38. 27449596 10.1016/j.drup.2016.06.001

[jkac188-B24] Wattam AR , Abraham D, Dalay O, Disz TL, Driscoll T, Gabbard JL, Gillespie JJ, Gough R, Hix D, Kenyon R, et al PATRIC, the bacterial bioinformatics database and analysis resource. Nucleic Acids Res. 2014;42(Database issue):D581–D591. 24225323 10.1093/nar/gkt1099 PMC3965095

[jkac188-B25] Zhang D , de Souza RF, Anantharaman V, Iyer LM, Aravind L. Polymorphic toxin systems: comprehensive characterization of trafficking modes, processing, mechanisms of action, immunity and ecology using comparative genomics. Biol Direct. 2012;7(1):18. 22731697 10.1186/1745-6150-7-18 PMC3482391

