# Supplementary material for: Characterization of the cluster MabR prophages of Mycobacterium abscessus and Mycobacterium chelonae
Source: G3 (Bethesda). 2022 Jul 27;12(9):jkac188. doi: 10.1093/g3journal/jkac188 (PMC9434293; doi:10.1093/g3journal/jkac188)
Supplement: jkac188_Supplementary_Figure_Legend [file jkac188_supplementary_figure_legend.docx]

**Figure S1** Genome organization of prophiFSAT01-1, prophiFSIL01-1, McProf, prophiFSQJ01-1 and prophiFVLQ01-1. The ruler represents the coordinates of the genome. Forward and reverse transcribed genes are shown as boxes above and below the ruler, respectively. Genes are colored according to their assigned Phamily with the Phamily number shown above each gene with the number of Phamily members in parentheses. Genome maps were generated using Phamerator and the database, “Actino_Mab_Draft (version 20).” (Cresawn et al. 2011)
